# A Network and Visual Quality Aware N-Screen Content Recommender System Using Joint Matrix Factorization

**DOI:** 10.1155/2014/806517

**Published:** 2014-04-03

**Authors:** Farman Ullah, Ghulam Sarwar, Sungchang Lee

**Affiliations:** Department of Information & Communication, Korea Aerospace University, Goyang 412-791, Republic of Korea

## Abstract

We propose a network and visual quality aware N-Screen content recommender system. N-Screen provides more ways than ever before to access multimedia content through multiple devices and heterogeneous access networks. The heterogeneity of devices and access networks present new questions of QoS (quality of service) in the realm of user experience with content. We propose, a recommender system that ensures a better visual quality on user's N-screen devices and the efficient utilization of available access network bandwidth with user preferences. The proposed system estimates the available bandwidth and visual quality on users N-Screen devices and integrates it with users preferences and contents genre information to personalize his N-Screen content. The objective is to recommend content that the user's N-Screen device and access network are capable of displaying and streaming with the user preferences that have not been supported in existing systems. Furthermore, we suggest a joint matrix factorization approach to jointly factorize the users rating matrix with the users N-Screen device similarity and program genres similarity. Finally, the experimental results show that we also enhance the prediction and recommendation accuracy, sparsity, and cold start issues.

## 1. Introduction

Multimedia streaming capable devices and social networks are growing rapidly. The convergence of broadband access networks, multimedia-capable devices, and social networks provide a platform to access multimedia content anytime and anywhere and to share the content and opinions with friends and other users. Smart mobile devices are influencing users to expect access to any content via his or her device. However, the heterogeneity of access networks and devices present new questions with respect to the user experiences and the quality of service of multimedia content on different devices. For instance, does a user enjoy and have the same experience of multimedia content in terms of visual quality when that content is viewed on two different devices—for example, a 21-inch television and a 10-inch tablet PC [1]?

The multiscreen report conducted by the Interactive Advertising Bureau (IAB) shows that in USA, 77% and 69% of people have three and four screens, respectively, to watch the content, but 65% of people say that they are unable to watch most of the same content on their smart phones [2]. In this paper, we propose a network and multimedia visual quality aware N-Screen content recommender system to personalize the contents with user preferences, and ensuring a good visual quality on the user N-Screen device with efficient available bandwidth utilization. We suggest an architecture to estimate the access network condition and find the available bandwidth for the content to be recommended. The proposed system recommends content that improves the user experience and provides better visual quality on his device; considering the current access network heterogeneity condition and device attributes.

Television is one of the most popular consumer products in every home around the world; evolved from analog to digital before entering the age of web-based TV. The bidirectional communication of the television enables the user to use the TV in more efficient and convenient ways. Web-based TV (Smart TV) enables users to collaboratively share knowledge about TV programs and provide feedback, which is used to personalize the content for users. The interactive services of the Smart TVs (STVs) and the social networks promote the recommender systems to help TV viewers to identify reliable and desirable programs from the abundance of options.

**Figure 1:**
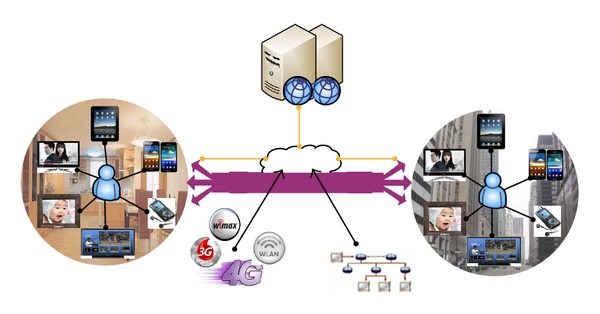
N-Screen (multiscreen) based content delivery system.

Figure 1 shows the model architecture of N-Screen (multiscreen) based content delivery system. N-Screen enables users to access the multimedia content from various sources and share the content through any device and from anywhere [3]. The recent explosion of smart devices and online multimedia streaming websites such as YouTube, Netflix, and Amazon video-on-demand provides more ways to interact with the multimedia services and watch the contents whether at home, at the office, or on the-go. We are now facing several challenges with the abundance of data and information. The users are not interested in wasting time to browse the programs due to the small screen size of mobile devices and limitation of wireless networks speed. The proposed system personalizes content considering user access network conditions and visual quality on his device with the user's preferences.

Recommender systems (RSs) are web-based applications, software tools, programs, and techniques providing suggestions for items/products to be of use to a user [1]. By suggesting TV programs that are most similar to user profiles, RSs have become the prominent tool to handle the information overload. RSs provide suggestions about items using either user feedback about previously experienced items or its description to find items similar to user usage profiles. In RSs, the core component is the recommendation algorithms that make decisions using various types of information related to users and items. Based on the algorithms of finding similarities and recommendations, the RSs can be classified as: content-based filtering (CBF), collaborative filtering (CF), and the hybrid approaches.

Most of the RSs use CF techniques [4–6], which predict and recommend an item to a user by using the users opinions about items to find the similar users and/or items. CF collects user opinions either explicitly using rating and feedback information or implicitly by mining the user's usage history patterns. Traditional CF methods only consider the user rating information without considering any other information. These methods need a large volume of history data to compute the similarity between users. The characteristic when the user rating increases poses critical issues on the data sparsity (fewer ratings and experienced programs) and the low accuracy of recommendations (due to sparsity and cold start issues).

Content-based filtering (CBF) recommends items that have similar content to the contents that the user had already experienced [7]. It uses item descriptions and metadata information [8] for finding similar users/items. CBF needs structural information of both the experienced and available programs. These methods do not consider the users opinion about contents and assume that similar programs are rated similarly. CBF has the limitation of a structural information requirement, useful for text-based recommendations but ineffective for unstructured items such as movies and music [6]. These filtering techniques restrict the user to only those types of programs that user previously experienced and cannot recommend programs with different features.

The hybrid approach [9] combines the CF and CBF to improve their limitations. Hybrid RSs either combine the CF and CBF or may combine any of them with any other information like user demographics. The age of Big Data, information retrieval and smart devices attract the researchers in the new area of recommendations considering the contextual information as temporal information [10], user mood based [11], and location-aware recommendations [12]. In this paper, we propose a novel recommender system that considers user access network heterogeneous nature of available bandwidth and visual quality on user N-Screen devices, in order to ensure that user device and access network are capable of streaming and displaying the content.

We organized the rest of the paper as follows. In Section 2, we provide background and related work about the available bandwidth and visual quality estimation, as well as the RSs.  Section 3 briefly describes our proposed architecture and recommendation scheme. In Section 4, we discuss the dataset and the results. Finally, we will conclude this paper and discuss future work in Section 5.

## 2. Background and Related Work

Before presenting the proposed RS, we first provide the background knowledge about the available bandwidth and visual quality estimation for users N-Screen devices. Then, we review the related works of RSs in view of the matrix factorization approach.

### 2.1. Available Bandwidth and Visual Quality Estimation

To provide the multimedia services efficiently to heterogeneous devices through different access networks, the streaming server should know the available bandwidth to support QoS (quality of service) for the streaming video. Significant work has been done to estimate the available bandwidth. The two existing methods for bandwidth estimation are the probe gap [13] and probe rate-based models [14]. The probe gap model finds the delay between the probe packets and infers the available bandwidth from the delay, while the probe rate based model finds the bandwidth by using a train of packets. The available network bandwidth is a time varying random variable that depends on the traffic load and heterogeneous access devices. Let *Bw*_*i*_(*t*, *T*) be the average available bandwidth of a link *i* over sometime period *T*, *C*_*i*_ is its capacity, and *λ*_*i*_(*t*) is the traffic load (used capacity) of the link at time *t*. Equation (1) finds the average available bandwidth of a link.

Consider

(1) $$\begin{array}{c} Bw_{i}\left(t,T\right)=\frac{1}{T}\overset{t+T}{\underset{t}{\int }}\left(C_{i}-\lambda_{i}\left(t\right)\right)dt. \end{array}$$

In the last decade, several research studies have been carried out to find the available bandwidth in congested networks to support the QoS. The packet train based probing methods are widely used for finding network utilization. Hu and Steenkiste in [15] proposed the initial gap increasing (IGI) and packet transmission rate (PTR) methods for finding the available bandwidth. The proposed techniques determine an initial packet pair gap that provides a high correlation on the bottleneck link and the packet gap at the destination. In probing methods, the packet train is a number of the same size packets transmitted back-to-back, called one way delay (OWD) based analysis model [16]. The delays of the consecutive probing packets and the probe packet size can be used to estimate the available bandwidth.

Multimedia over mobile networks is a booming industry these days. In order to provide these services efficiently, the most vital requirements are the available bitrates, the frame rate of multimedia content, the user's device screen size, and the resolution. The visual quality estimates the expected user opinion regarding the multimedia content visual quality on the device. To predict the visual quality, different evaluations and standardized efforts have been made. The visual quality models can be classified as (1) media layer models analyze the video content to predict the video quality and (2) packet layer models consider IP-Packet information in terms of packet loss or use the packet payload to extract some media information.

ITU-T J.144 [17] and ITU-R BT.1683 [18] are standards for estimating the visual quality in digital television applications, when the original reference signal is available. These standards can be used for a full reference model where the received noisy signal is compared with the original signal. ITU-T G.1070 [19] is a standard for visual perceptual quality estimation in an IP-Packet network considering the packet loss and device information. Catellier et al. [20] explored and evaluated the audiovisual quality impact on different mobile devices. They considered the video resolution, viewing distance, and screen size for estimating the video quality rating. A parametric model is proposed in [21] to provide perceptual quality estimation for multimedia contents coded with different codecs, bit rates, and display formats.

### 2.2. Recommender (Content Personalization) Systems

The rapid development of smart devices and communication technologies has provided people with convenient access to content from anywhere, but users are simultaneously suffering from information overload. Nowadays, users have greater access to massive amounts of data from numerous sources, which leads to the issue of finding the most relevant information. The recommender systems help users to find relevant items by allowing the system to utilize users profiles [1]. The central part of the recommender systems is the decision making algorithms to find the relevant items. The recommender systems can be classified as collaborative filtering, content-based filtering, and hybrid filtering.

Collaborative filtering (CF) methods utilize user's opinions about programs to find similar users/items to the target user and use their programs as candidates for recommendations. These methods need a large volume of history data for computing the similarity between users. The computational complexity increases as the rating data increases. Based on computational complexity, the CF can be divided into memory-based CF and model-based CF [10]. Memory-based CF uses the entire history data to find similar users and items to use it as a candidate for recommendations to the target user. These methods have the issue of finding similar users, when users have less and sparse ratings information. Memory-based CF has the advantage of high accuracy but at the cost of high computation. The model-based CF approach performs precomputation to extract some features from the history data and learns a model based on these features that can be used instead of the whole dataset.

In the model-based approach, matrix factorization is an efficient and effective recommendation scheme used to factorize the users rating matrix into user-specific and item-specific matrices and use these matrices for further missing information prediction [22–24]. The singular value decomposition (SVD) [23] decomposes a matrix like *R*_*M*×*N*_ where *M* and *N* are the number of users and items, respectively, into three matrices:

(2) $$\begin{array}{c} R_{M\times N}=U_{M\times r}\Sigma_{r\times r}V_{N\times r}^{T}. \end{array}$$

The low rank matrix factorization method [24] approximates the multiplication of *r*-rank factorization matrices:

(3) $$\begin{array}{c} R≅U^{T}V. \end{array}$$

In matrix decomposition, *R* is a dense matrix and SVD approximates the matrix *R* by

(4) $$\begin{array}{c} \underset{U,V}{\min}\frac{1}{2}{||R-U^{T}V||}_{F}^{2}, \end{array}$$

where ||·||_*F*_^2^ is the Frobenius norm. Since the users rating matrix is very sparse, (4) cannot be applied directly, so the rating-matrix *R* can be approximated by using (5) proposed by Koren et al. in [25].

Consider

(5) $$\begin{array}{c} \underset{U,V}{\min}\frac{1}{2}\overset{M}{\underset{m=1}{\sum }}\overset{N}{\underset{n=1}{\sum }}I_{mn}{\left(R_{mn}-U_{m}^{T}V_{n}\right)}^{2}+\frac{\lambda }{2}\left({||U||}_{F}^{2}+{||V||}_{F}^{2}\right), \end{array}$$

where *I*_*mn*_ is 1, if the item *n* is rated by the user *m* and (*λ*/2)(||*U*||_*F*_^2^ + ||*V*||_*F*_^2^) is a regularization factor to avoid the over fitting.

Content-based filtering (CBF) recommends items having similar content features to the programs that users have previously experienced. It uses the description and metadata information of programs to find similar users. The content-based filtering has the issues of structural information requirements, content description, and similarity issues of a new user. Collaborative filtering has issues of sparsity, cold start, and scalability. The hybrid recommender systems combine the collaborative filtering and content-based filtering or either some features of both filtering techniques.

The contribution points of the paper can be summarized as follows. (1) The proposed recommender system estimates the user available access network traffic and visual quality on the user's N-Screen device to ensure a better visual quality with efficient bandwidth utilization. (2) We suggest a joint matrix factorization approach to incorporate the rating matrix, N-Screen device similarity (DS) in terms of available user access network bandwidth and visual quality and the program genre-based similarity (GS) to improve the accuracy, precision, and recall. (3) We estimate the visual quality on the user's device to recommend device capable content recommendations. (4) It ensures that the access network is capable of streaming the recommended content and will produce good visual quality on the user's device. (5) The proposed system is more appropriate for online movies, video-on-demand services, IPTV, and Smart TV programs recommendations to user N-Screen devices.

## 3. The Proposed Network and Visual Quality Aware N-Screen Content Recommender System

**Figure 2:**
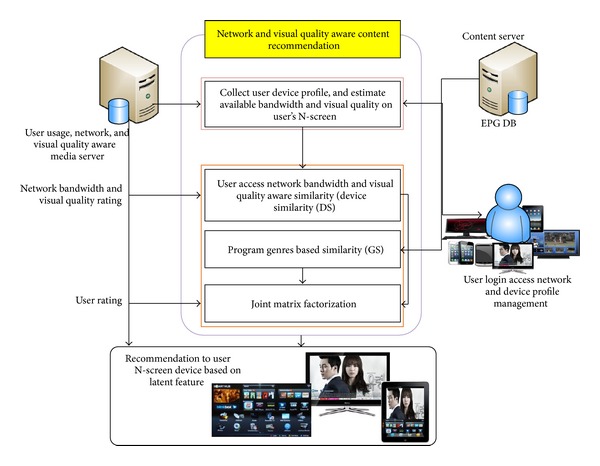
Architecture of proposed network and visual quality aware N-Screen content recommender system.

In this section, we present the proposed architecture of the N-Screen content recommender system.  Figure 2 shows the architecture of the proposed system. When a user logs into the system, it acquires the user device profile and estimates the available bandwidth and visual quality on the device. We focus the discussion on two key components: (1) available bandwidth and visual quality estimation on the user's N-Screen devices and (2) components of the recommendation algorithm, namely, the network (available bandwidth) and video quality aware similarity (device similarity), program genres-based similarity, and the joint matrix factorization model to jointly factorize these similarities with the rating matrix.

### 3.1. Available Bandwidth and Visual Quality Estimation on User N-Screen Devices

**Figure 3:**
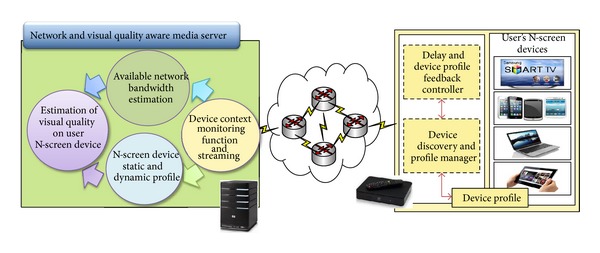
Architecture of network and visual quality estimation on user's N-Screen device.

Available bandwidth and visual quality are critical factors to provide QoS in N-Screen services. In N-Screen, a user can access multimedia contents through multiple devices having different attributes including codecs, screen sizes and resolutions, and CPUs and through different access network interfaces such as fixed networks, 3G/4G and Wi-Fi [3].  Figure 3 shows the architecture for available bandwidth and visual quality estimation on the user's device. Available bandwidth estimation in a congested network is helpful to estimate visual quality on the user device to improve the user experience of content consumption. The prominent approaches for available bandwidth estimation are the packet pair-probe scheme and packet train scheme. In this paper, we propose the device context monitoring function at the server side and the delay and device profile feedback controller at the setup box to estimate the available bandwidth. The device context monitoring sends bunches of packet trains back-to-back to estimate the available bandwidth. The feedback controller time stamps the packet (the receiving time of the packet) and sends it in ACK to the device context monitoring system.

**Figure 4:**
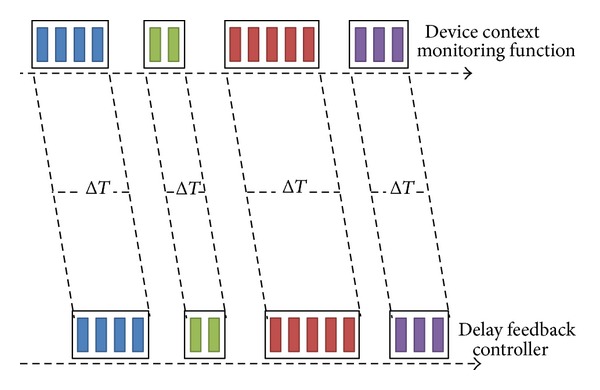
Available bandwidth estimation using packet train scheme.

Figure 4 shows the pictorial representation of the delay and available bandwidth estimation scheme. Let *d* be the size of the probe packet in the packet train and let Δ*T* be the total time for the packet train. Equation (6) can estimate the available bandwidth.

Consider

(6) $$\begin{array}{c} Bw_{i}=\frac{\sum_{j=1}^{N}d_{j}}{\Delta T}, \end{array}$$

where *Bw*_*i*_ is the available bandwidth estimated for the packet train *i* and *N* is the number of probe packets in the packet train. The device context monitoring function transmits the packet-train at variable rates to estimate the variation in the available bandwidth and use the average normalized value for network preference (available bandwidth) for that program.

The good quality of visual effects on the user's device can increase the user's interest in the content. The most reliable method for estimating visual quality is to provide original and degraded videos to different viewers and get an average opinion. The metrics used for the evaluation of the visual quality is the Mean Opinion Score (MOS) or the difference mean opinion score (DMOS). We suggested to use the parametric model [21] for estimation of the perceptual visual quality, that is, MOS on the user device by using

(7) $$\begin{array}{c} \text{MOS}=v_{3}\left(1-\frac{1}{1+{\left(Bw/v_{4}\right)}^{v_{5}}}\right), \end{array}$$

where MOS is the multimedia visual quality on a user's device in the range of (1–5), *Bw* is the available bandwidth, and *v*_3_, *v*_4_, and *v*_5_ are the model parameters that are dependent on the codec, frame rate, and video screen size.

### 3.2. The Proposed Recommendation Scheme

**Figure 5:**
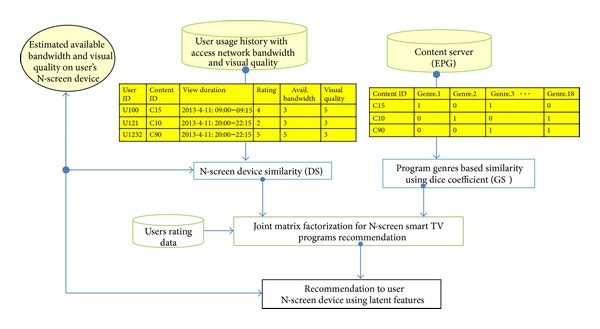
Algorithm for proposed network and visual quality aware N-Screen content recommendation.

In this subsection, we present the propose scheme of N-Screen content recommendation.  Figure 5 shows the algorithm of the proposed recommendation scheme. The recommendation scheme is based on the user's N-Screen device similarity (DS), genre-based similarity (GS), and the joint matrix factorization to combine these similarities with the rating matrix in the factorization model.

#### 3.2.1. N-Screen Device Available Bandwidth and Visual Quality Similarity (Device Similarity)

In Section 3.1, we explained the architecture and procedure of finding the available bandwidth and visual quality on a user's device. Using (6) we estimate the available and required bandwidth and find the network preferences for the content. Equation (7) estimates the visual quality on the user's device considering the available bandwidth and device attributes, and assigns the MOS value. Using the available bandwidth and the visual quality, we find the user N-Screen device similarity (DS) by cosine similarity function using

(8) $$\begin{array}{c} \text{D}{\text{S}}_{ij}=\frac{\sum_{k=1}^{K}P_{ik}D_{jk}}{\sqrt{\sum_{k=1}^{K}P_{ik}^{2}}\sqrt{\sum_{k=1}^{K}D_{jk}^{2}}}, \end{array}$$

where DS_*ij*_ represents the similarity between the user's current access network available bandwidth and visual quality on the user's N-Screen device with the database of the movies that contains the history for access network speed and visual quality of different users experienced programs on various device. *P* shows the vector of estimated values of the available bandwidth and the visual quality on user's N-Screen device and access network, and *D* is the user movie network bandwidth and visual quality of the experienced programs by different users on different devices.

#### 3.2.2. Genre-Based Programs Similarity Using Dice Coefficient

We define the similarity between programs genres using the dice coefficient. Each program has different genres (Drama, Comedy, Thrill, etc.), and we represent the genres in binary vector form *G* = {0, 1, 0, 0, 1, 0, 0,…, 1}. Using (9), we find the similarity between different programs:

(9) $$\begin{array}{c} \text{G}{\text{S}}_{ij}=\frac{2\sum_{k_{1}}^{K_{1}}G_{ik_{1}}G_{jk_{1}}}{\sqrt{\sum_{k_{1}}^{K_{1}}G_{ik_{1}}^{2}}+\sqrt{\sum_{k_{1}}^{K_{1}}G_{jk_{1}}^{2}}}, \end{array}$$

where GS_*ij*_ represents the genre-based similarity between two programs, *K*_1_ is the total number of genres, and *k*_1_ is a genre in a program.

#### 3.2.3. Joint Matrix Factorization for N-Screen Content Recommendation

In Section 2.2, (5) represents the matrix factorization model. In the model, *R* shows the user-item rating matrix, *M* is the number of users, and *N* is the number of programs, respectively. *U* and *V* are the users and items specific latent features. The user experience with the content increases if it produces good visual quality on user's device. Based on this intuition and also the high speed motion videos that need higher bandwidth, we suggest a joint matrix factorization model that incorporates the users device similarity, the genre-based similarity, and the users rating matrix. We extend the joint matrix factorization approach proposed in [26]. Equation (10) shows the joint matrix factorization model for the N-Screen content recommendation:

(10) min⁡U,VL(U,V)=12∑m=1M∑n=1NImn(Rmn−UmTVn)2 +α2∑n=1NIcn(DScn−1|Γ(n)|∑jεΓ(n)VnTVj)2 +β2∑n=1N∑j=1N(GSnj−VnTVj)2 +λ2(||U||F2+||V||F2),

where |Γ(*n*)| represents the number of top-most similar content to *V*_*n*_, *α* and *β* are the weighting parameters to regularize the contribution of the device-specific and the genre-based similarities respectively. The value of *α* and *β* should be greater than zero to contribute to the objective function (10). The *I*_*cn*_ denotes the indicator function if the program *n* experienced network preference and visual quality similarity with the current device available bandwidth and visual quality, so it will be 1 if DS_*cn*_ > 0; otherwise, it will be 0. Also *I*_*nj*_ is 1 if GS_*nj*_ > 0 and 0 otherwise.

In the objective function of (10), we introduce the user device specific similarity as a regularization term:

(11) $$\begin{array}{c} \frac{\alpha }{2}\overset{N}{\underset{n=1}{\sum }}I_{cn}{\left(\text{D}{\text{S}}_{cn}-\frac{1}{\left|\Gamma (n)\right|}\sum_{j\varepsilon \Gamma (n)}V_{n}^{T}V_{j}\right)}^{2}. \end{array}$$

In (11), we improve the content visual quality by considering most similar programs that have better visual quality on a user's device. We also consider the genre-based similarity as a regularization term by the intuition that similar genres have the same requirement of network bandwidth and visual quality on user's N-Screen device. A program having the genre sport have high speed motion and need more bandwidth. Equation (12) shows the regularization term for the genre-based similarity in the whole objective function

(12) $$\begin{array}{c} \frac{\beta }{2}\overset{N}{\underset{n=1}{\sum }}\overset{N}{\underset{j=1}{\sum }}{\left(\text{G}{\text{S}}_{nj}-V_{n}^{T}V_{j}\right)}^{2}. \end{array}$$

The joint matrix factorization model incorporates the N-Screen device and the genre-based similarities in the objective function. Considering the DS, it recommends content that the user's current access network is capable of streaming and provides better visual quality on a user's device. We also improve the cold start issues by considering the device similarity and the genre-based similarity with the users rating matrix. We perform the gradient of (10), with respect to *U*_*m*_ and *V*_*n*_ to achieve the local minimum of the objective function

(13) ∂L(U,V)∂Um=∑n=1NImn(UmTVn−Rmn)Vn+λUm,∂L(U,V)∂Vn=∑m=1MImn(UmTVn−Rmn)Um +2α1|Γ(n)|∑jεΓ(n)Icn(VnTVj−DScn)∑jεΓ(n)Vj +2β∑j=1NInj(VnTVj−GSij)Vj+λVn.

We update iteratively the matrices *U* and *V* by the gradient descent using

(14) $$\begin{array}{c} U^{\left(t+1\right)}=U^{t}-\eta \frac{\partial L^{t}\left(U,V\right)}{\partial U^{t}},\\ V^{\left(t+1\right)}=V^{t}-\eta \frac{\partial L^{t}\left(U,V\right)}{\partial V^{t}}, \end{array}$$

where *η* the gradient descent function is learned iteratively to converge the gradient function and *t* shows the iteration number. The initial values of *U* and *V* are randomly selected and updated at each iteration. We update the *L* ^*t*^(*U*, *V*) using (10), and iterate until the *L* ^*t*^(*U*, *V*) converged to the threshold or the specified number of iterations achieved.

## 4. Simulation and Experimental Results

In this section, we briefly describe the dataset and experimental results we achieved. First, we will explain the performance comparison of the available network bandwidth and visual quality on multiple (N-Screen) devices, and then we will describe the results of the proposed N-Screen content recommender system.

### 4.1. Performance Comparison of Available Network Bandwidth, Frame Rate, and Visual Quality on User N-Screen Devices

**Figure 6:**
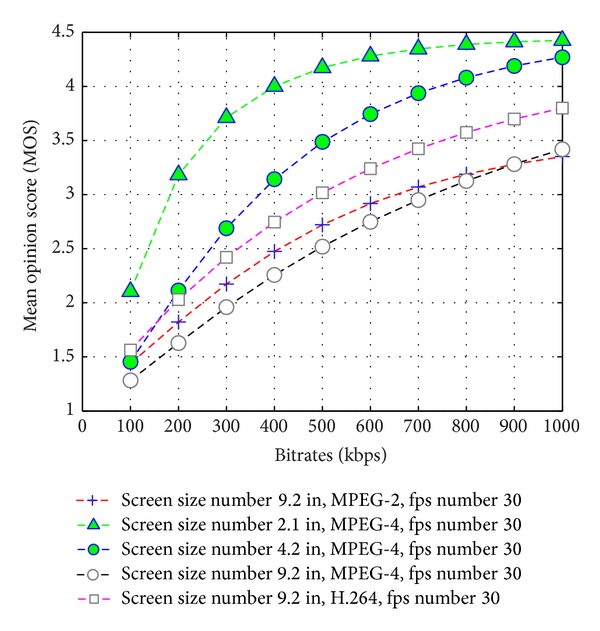
Estimated video quality on N-Screen devices versus bitrates.

**Figure 7:**
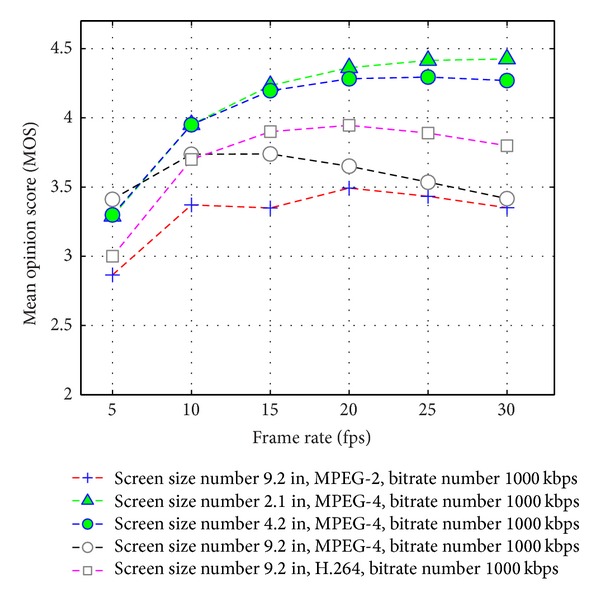
Estimated video quality on N-Screen devices versus frame rate.

In N-Screen services, a user can access multimedia content on different devices having different codecs, frame rates, screen sizes, resolution, and access network throughput (bitrate). We evaluate the performance comparison of video quality on user N-Screen devices with available bandwidth, frame rate, and packet loss ratio (PLR) using [27].  Figure 6 shows the performance comparison of the estimated video quality with respect to the available bandwidth. The interpretation of the MOS values show that we need higher bandwidth as the screen size increases with the same media codec and the frame rate. In order to improve, the user experience with the content we should estimate available bandwidth and recommend those contents that user access network and device are capable of streaming with user preferences.  Figure 7 shows the visual quality comparison with the frame rate. The graphs show that as the screen size increases at a low bitrate, we are not able to provide higher frame rate multimedia content. To improve the user experience with the content and seamless service continuity to the user's N-Screen devices, it necessitate a recommender system that considers the content genre information to recommend high speed motion multimedia on higher frame rate and network speed (available bandwidth) capable device.

**Figure 8:**
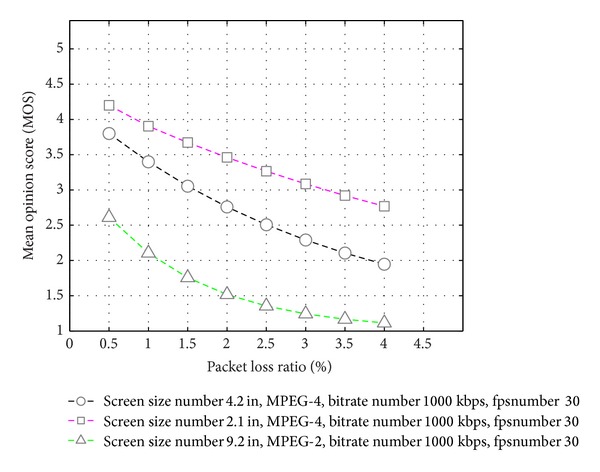
Estimated video quality on N-Screen devices versus packet loss ratio (PLR).

Figure 8 shows the performance comparison of the visual quality with respect to the packet loss in the streaming (access) network. The graphs show that the PLR has a diverse effect over the visual quality on large screens. In an N-Screen service, a user can watch the content at different locations and times through heterogeneous access networks (wired/wireless), so the access network has different PLR at different locations. Analyzing the video quality MOS over N-Screen devices at different bit rates, frame rates, and packet loss ratio, it is clearly seen that we need a recommender system that personalizes the N-Screen content so that the user can watch the content anytime and anywhere with his preferences.

### 4.2. Evaluation of the Proposed Network and Visual Quality Aware N-Screen Content Recommender System Using Joint Matrix Factorization

In this section, we will explain the dataset used for the evaluation of the proposed system and the simulation results achieved during the evaluation. We performed several experiments and compared the proposed system with the well-known recommendation methods and systems.

#### 4.2.1. Dataset

We conducted our experiments on the dataset, retrieved from the Yahoo! Movies [28]. It is a multicriteria dataset and users provide preferences on four criteria: acting (*A*_1_), story (*A*_2_), direction (*A*_3_), and visuals (*A*_4_). To the best of our knowledge, currently there is no dataset that have the user N-Screen attributes, available bandwidth, and visual quality. The Yahoo! dataset have the visual quality only, so we modified the Yahoo Movies dataset accordingly by inserting the various bitrates and corresponding visual quality. Users provided preferences values with *A*+ denoting the most preferred and *F* is the least preferred. In the dataset, users also provided the overall preferred value over the movie, and we convert the dataset in the range of (1–5). In short, the dataset we used included the user preferences, available bandwidth (bitrates/throughput) and visual quality of user N-Screen devices and genres (18-genres) of the movies.  Table 1 shows the brief description of the user-movies rating matrix (dataset).

We randomly used 80% of the training data and 20% of the testing data independently, and performed 10-fold cross-validation of the proposed system.

#### 4.2.2. Metrics

For the evaluation of the proposed system, we used the two most common metrics: (1) mean absolute error (MAE) and (2) precision at top-*N* recommendation list (*P*@*N*) [29, 30]. MAE measures the prediction quality of the recommender system with the lowest value of MAE having better prediction quality. The MAE metric can be measured as

(15) $$\begin{array}{c} \text{MAE}=\frac{1}{N}\sum_{u,j}\left|R_{uj}-\hat{R_{uj}}\right|, \end{array}$$

where *N* is the number of ratings in the test dataset, *R*_*uj*_ is the actual rating of user *u* on item *j*, and $\hat{R_{uj}}$ is the predicted rating. Precision and recall are the most common measures used in information retrieval systems and search engines to measure the accuracy. Precision is the fraction of relevant items/programs out of retrieved programs for a query. It assigns equal weights to the Top-*N* most relevant items so we use the precision at *N* (*P*@*N*). The (*P*@*N*) is the average ratio of the number of relevant programs over the ranked list (Top-*N*) recommended programs for users in the test dataset then *P*@*N* is

(16) $$\begin{array}{c} P@N=\frac{r}{N}, \end{array}$$

where *r* is the relevant programs in the recommended Top-*N* (at ranked *N*) programs.

#### 4.2.3. Impact of Tradeoff Parameters *α* and *β*

In the suggested joint matrix factorization, the parameters *α* and *β* control the contribution of device similarity (DS) and TV program genre similarity (GS), respectively. If the values of *α* and *β* are very small, then we have to mine only the user rating matrix. On the other hand, if the values of *α* and *β* are large, the similarities DS and GS dominate the user rating. We analyzed the values of *α* and *β* considering the different latent features dimensions and analyzed how they affected recommendation accuracy MAE.

**Figure 9:**
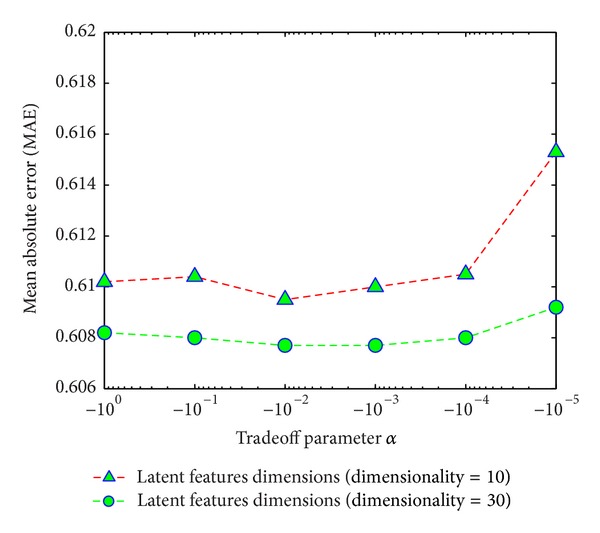
Impact of tradeoff parameter *α* with different dimensions of latent features.

**Figure 10:**
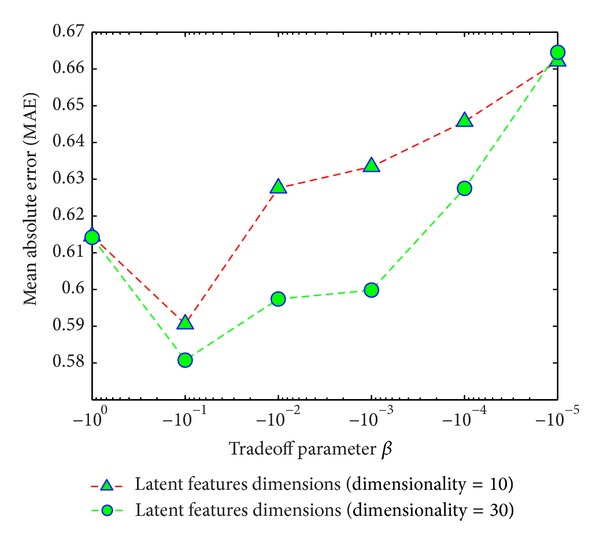
Impact of tradeoff parameter *β* with different dimensions of latent features.

Figures 9 and 10 show the impact of the tradeoff parameters *α* and *β* on recommendation accuracy by considering different dimensions of latent features. The figures clearly show that at different dimensions, when the values of *α* and *β* go down, the prediction accuracy increases (MAE value decreases). However, after a certain limit the value of MAE become increasing. We use the learning rate *η* = 0.001, and from the figures, it is clearly shown that the most suitable values for *α* = 0.01 and *β* = 0.1.

#### 4.2.4. Performance Comparison of Proposed N-Screen Content Programs Recommender System

We compared our proposed recommender system with the state of the art approaches and methods of recommender system. To the best of our knowledge the only recommender system that considers the user device composition (attributes) is proposed in [31], but they just provided the architecture and not simulation results. We compare our proposed recommender system with the following.

*Item-Based Collaborative Filtering* [32]. The item-based methods focus on the user experienced items and assume that similar items are rated similar. They use the user rating matrix and find item-item similarity using cosine similarity approach.

*Matrix Factorization Approach (MF)* [25]. This method is proposed by Koren et al. They use the user rating matrix and factorize it into user features matrix *U* and item feature matrix with multiple features, and use the objective function as shown in (5).

*Probabilistic Matrix Factorization (PMF)* [33]. The approach is proposed by Salakhutdinov and Mnih. They use the user rating matrix and the probability density function of the Gaussian distribution to factorize it into users and items feature specific matrices, respectively.

**Figure 11:**
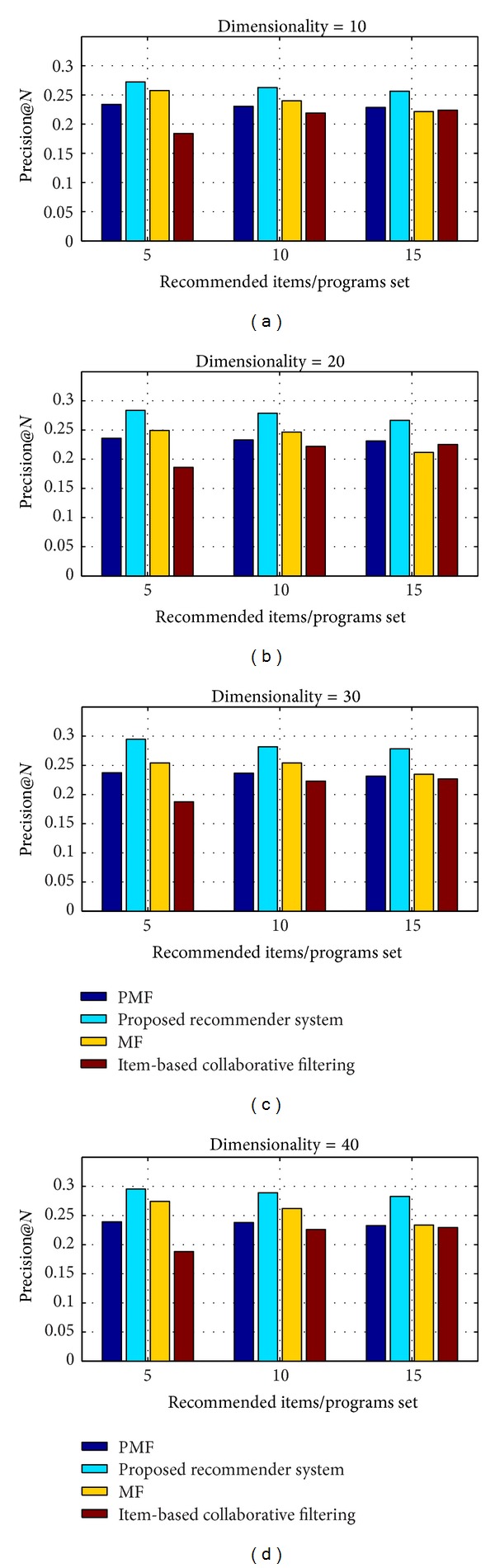
Comparison of precision @*N* (*P*@*N*) at different dimension of latent features.

**Figure 12:**
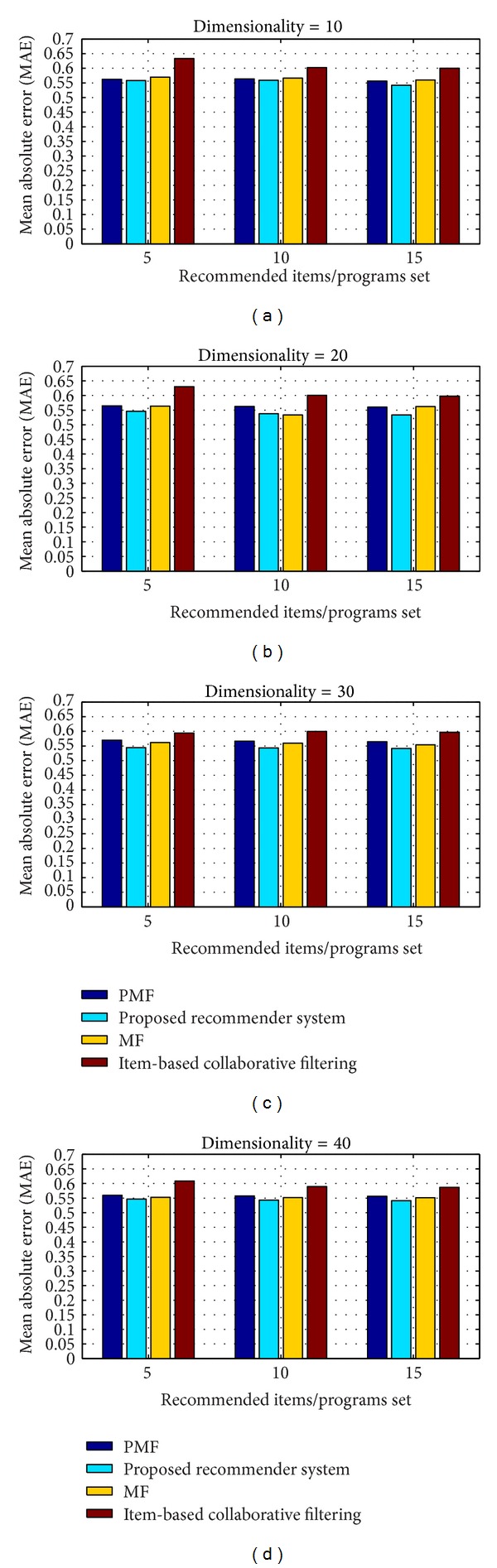
Comparison of MAE of *P*@*N* at different dimension of latent features.

We compare and analyze the proposed system with the state of the art methods in terms of *P*@*N* and MAE. We analyze the *P*@5, *P*@10, and *P*@15 at different dimensions of latent features 10, 20, 30, and 40.  Figure 11 shows the comparison of the *P*@*N*, and the system having high value means good recommendation accuracy. The figure clearly shows that our proposed network and visual quality aware system improve the *P*@*N* value as compared to the traditional cosine similarity approach (item-based collaborative filtering) and the matrix factorization approaches. The MAE value shows the prediction accuracy of the system and having low MAE value means better accuracy of recommendation.  Figure 12 shows the MAE performance comparison of *P*@*N* by recommending programs using different latent features dimensions. The proposed system has low MAE value compared to the other baseline methods and recommendation approaches.

## 5. Conclusion

This paper presents a novel N-Screen content recommender system that ensures a visual quality on user's N-Screen devices and effective access network available bandwidth utilization with his content preferences. In N-Screen, a user can access multimedia contents through multiple devices having various attributes like codecs, screen sizes, CPUs, and heterogeneous access networks and network conditions at different locations and times. The simulation results of available bandwidth and visual quality clearly show the issues of providing the QoS on user N-Screen devices and necessitate a recommender system to personalize the user N-Screen content with his preferences. The proposed system guarantees that the user access network and N-Screen device are capable of displaying and streaming by incorporating the estimated available bandwidth and visual quality with users rating and programs genre information. Furthermore, we introduce a delay and device profile feedback controller at the user's N-Screen device and device context monitoring function at the media server to send a packet-train at a variable rate to estimate the available bandwidth and visual quality. We integrate the programs genre considering that it affects the available bandwidth and the visual quality, for example, a program having a sport genre needs a higher bandwidth and frame rate. In addition, the joint matrix factorization improves the prediction and recommendation accuracy by jointly factorizing the users rating information with the user device similarity in terms of available bandwidth and visual quality and program genres similarity. Finally, with the user preferences the proposed recommender system ensures that user access network and N-Screen device are capable of streaming and display.

## Figures and Tables

**Table 1:** Statistics of Yahoo! Movies dataset.

Dataset attributes	Statistics
Number of users in training dataset	7,642
Number of movies in training dataset	11,915
Total number of ratings in training dataset	211,231
Average ratings per user in training dataset	27.64
Average ratings per movie in training dataset	17.73
Number of users in test dataset	2,309
Number of movies in test dataset	2,380
Total number of ratings in testing dataset	10,136
Average number of ratings per user in test dataset	4.39
Average ratings per movie in test dataset	9.54
Genres of movies data	18
