# Erratum to “A Network and Visual Quality Aware N-Screen Content Recommender System Using Joint Matrix Factorization”

**DOI:** 10.1155/2014/859292

**Published:** 2014-12-29

**Authors:** Farman Ullah, Ghulam Sarwar, Sungchang Lee

**Affiliations:** Department of Information & Communication, Korea Aerospace University, Goyang 412-791, Republic of Korea

In “A Network and Visual Quality Aware N-Screen Content Recommender System Using Joint Matrix Factorization,” there was an error of the negative sign on the *x*-axis in Figures 9 and 10. The right figures are provided as follows.

## Figures and Tables

**Figure 9:**
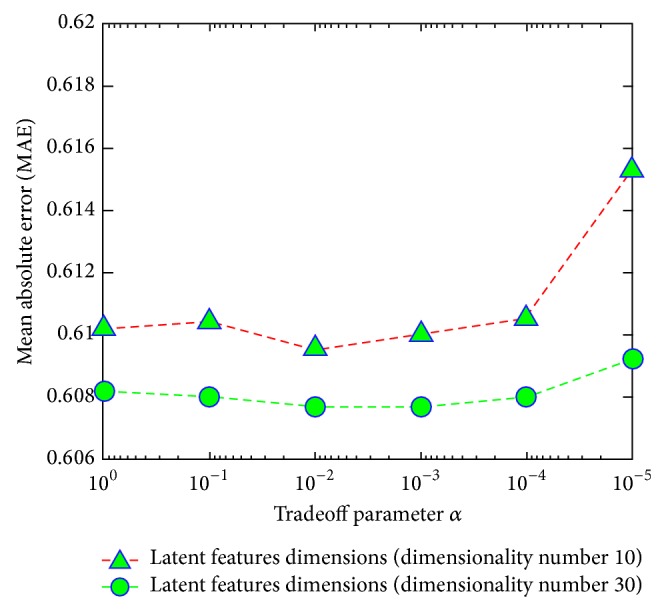
Impact of tradeoff parameter *α* with different dimensions of latent features.

**Figure 10:**
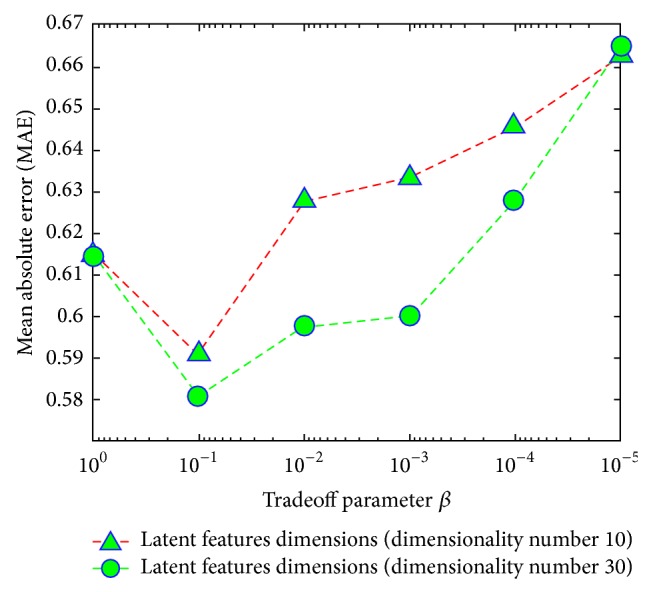
Impact of tradeoff parameter *β* with different dimensions of latent features.

